# Editorial: Exercise in Pediatric Medicine

**DOI:** 10.3389/fped.2020.00476

**Published:** 2020-09-16

**Authors:** Tim Takken

**Affiliations:** Department of Exercise Physiology, Child Development & Exercise Center, Wilhelmina Children's Hospital, University Medical Center Utrecht, Utrecht, Netherlands

**Keywords:** fitness, exercise is medicine, exercise testing, exercise training, sport, physical activity, health promotion

Physical activity and exercise have a high potential in the treatment and prevention of many chronic medical conditions (CMC). Recent statistics from the Netherlands indicate that 1 in 10 children have a CMC, with a higher number in North America, where about 1 in 6 children are estimated to have a CMC. These children and their families often live complicated lives and are, in general, more frequent users of the healthcare system. Participation in organized and non-organized physical activities and sports is often more challenging for these children, as illustrated in the first ever Netherlands Physical Activity Report Card + for youth with a CMC (Burghard et al.).

Sufficient, age-appropriate levels and types of activities are important for healthy social, psychological, and physical development. The complicated relationship between physical activity and motor competence in pre-schoolers was also reported by Schmutz et al..

Alias et al. showed that the majority of children with Acute Lymphoblastic Leukemia (ALL) had low levels of daily physical activity after intensive chemotherapy. The authors recommend that physical activity should thus be promoted during and after cessation of ALL treatment to prevent long-term health risks and improve the overall quality of life (Alias et al.).

In subjects with clinically stable Cystic Fibrosis (CF), expiratory muscle strength might be a limiting factor for exercise performance. Sovtic et al. found an association between this factor and a decrease in exercise performance. They suggest that an increase in expiratory muscle strength might result in the improvement of exercise tolerance, a subject that needs further research (Sovtic et al.).

## Intervention Programs

There are several publications on this topic describing intervention studies. We have to be aware that performing intervention studies on a group of children with CMC is often challenging and very difficult. The authors of these studies are congratulated on their work. The inclusion of “real world” interventions and web-based interventions are also very welcome. Lakes et al. described the effects of therapeutic ballet intervention in children with Cerebral Palsy. They observed increased physiological and cognitive functions in the children included in the study (Lakes et al.) Another report by Gitimoghaddam et al. described the effects of a pilot study investigating gymnastic-based movement therapy in children with neurodevelopmental disabilities. The study was undertaken in a natural environment and discusses the challenges faced by researchers investigating this kind of program (Gitimoghaddam et al.).

Zwinkels et al. reported that anaerobic performance and fat mass improved following a 6 month school-based sports program in children with CMC. These effects are promising for long-term fitness and health promotion because school-based interventions eliminate many barriers to the implementation of sports programs in this population (Zwinkels et al.).

Morales et al. performed a secondary analysis to identify factors that explain training effects in improvement in pediatric cancer patients and observed considerable individual variability. The biggest improvements were found in those with the lowest baseline fitness levels. This study is the first attempt to personalize exercise prescription in pediatric cancer patients (Morales et al.).

Lu et al. undertook a school-based exercise intervention program for children with asthma from a predominantly economically disadvantaged and minority population. They reported good feasibility and adherence to the program. Furthermore, the intervention was effective in improving aerobic fitness, body composition, asthma quality of life, and lipid outcomes. These data indicate the need for a larger multicenter trial designed to study “exercise in pediatric medicine” in this population (Lu et al.).

Another study by Atalla et al. presented real-world data on the intervention effects of physical activity and sedentary behavior among 3592 young people in a Brazilian city. Although there was no overall effect, physically inactive individuals had increased PA levels, and overweight participants with obesity experienced a reduction in BMI *z*-score. This finding reiterates the value of these types of interventions in increasing the health of a large group of children (Atalla et al.).

A novel way to deliver exercise interventions is by using a tele-exercise platform. Chen et al. reported on the use of an online platform to deliver supervised virtual group exercise in children with Cystic Fibrosis. They showed that the children had a positive experience with optimal participation and no risk for cross-infection: a very promising approach for delivering therapeutic exercise children with Cystic Fibrosis (Chen et al.).

Lastly, a contribution to this issue discusses the design of a study to investigate the effects of a web-based exercise intervention in children with congenital heart disease (Meyer et al.). This is also a promising approach to deliver therapeutic exercise to children who often have to travel a long distance to the hospital. Web-based interventions are more feasible in these populations than center-based interventions (Meyer et al.).

## Sports

### Several Studies Reported on Sport-Specific Topics

DiCesare et al. found that sport-specialized female athletes have biomechanical changes during puberty that are indicative of potentially compromised neuromuscular control that may increase their risk of injury. Consideration of maturation status may be an important factor in assessing the injury risk profiles of adolescent athletes who specialize in a sport (DiCesare et al.).

Lammers et al. investigated exercise-induced bronchoconstriction (EIB) in a pediatric population. They reported that predictions of EIB by pediatricians are sensitive but not specific and that the prediction of EIB severity was poor, with very important findings for awareness of disease management in children with EIB (Lammers et al.).

Other children with exercise-induced complaints are patients with exercise-induced laryngeal obstruction. Olin describes the preferred diagnostic and therapeutic approaches in a review article. As current diagnostic technology has considerably improved in recent years, more evidence-based approaches to treatment are needed (Olin).

Many pediatric patients with CMC are receiving suboptimal treatment in physical activity and fitness because many clinicians might not have adequate knowledge when it comes to physical activity counseling, testing, and training. With this Research Topic in Frontiers in Pediatrics, we hope that a part of this gap is filled.

## Author Contributions

The author confirms being the sole contributor of this work and has approved it for publication.

## Conflict of Interest

The author declares that the research was conducted in the absence of any commercial or financial relationships that could be construed as a potential conflict of interest.

